# A Biophysics Toolbox for Reliable Data Acquisition and Processing in Integrated Force–Confocal Fluorescence Microscopy

**DOI:** 10.1021/acsphotonics.3c01739

**Published:** 2024-03-18

**Authors:** Zhaowei Liu, Edo van Veen, Humberto Sánchez, Belén Solano, Francisco J. Palmero Moya, Kaley A. McCluskey, Daniel Ramírez Montero, Theo van Laar, Nynke H. Dekker

**Affiliations:** † Department of Bionanoscience, Kavli Institute of Nanoscience, Delft University of Technology, 2629 HZ Delft, The Netherlands; ‡ Clarendon Laboratory, Department of Physics, University of Oxford, Oxford OX1 3PU, U.K.; § Kavli Institute of Nanoscience Discovery, University of Oxford, Dorothy Crowfoot Hodgkin Building, Oxford OX1 3QU, U.K.

**Keywords:** optical tweezers, force spectroscopy, fluorescence spectroscopy, data analysis, automated data acquisition, protein−DNA interactions

## Abstract

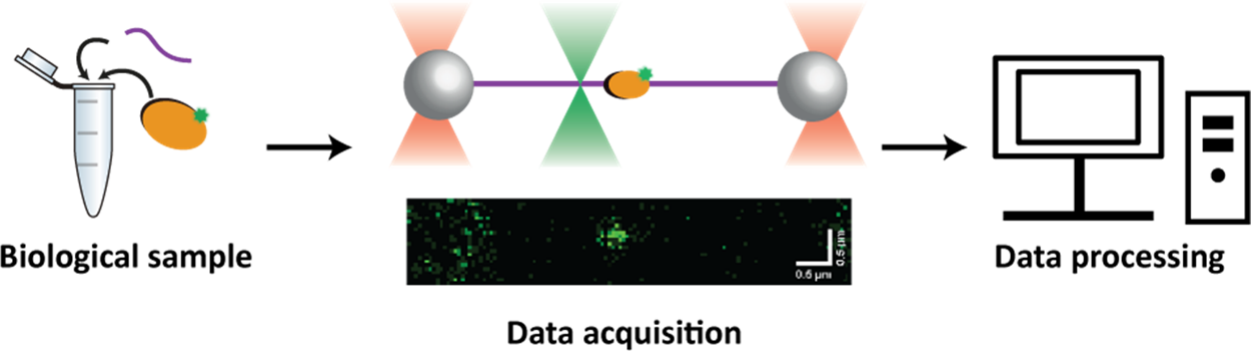

Integrated single-molecule force–fluorescence spectroscopy setups allow for simultaneous fluorescence imaging and mechanical force manipulation and measurements on individual molecules, providing comprehensive dynamic and spatiotemporal information. Dual-beam optical tweezers (OT) combined with a confocal scanning microscope form a force-fluorescence spectroscopy apparatus broadly used to investigate various biological processes, in particular, protein:DNA interactions. Such experiments typically involve imaging of fluorescently labeled proteins bound to DNA and force spectroscopy measurements of trapped individual DNA molecules. Here, we present a versatile state-of-the-art toolbox including the preparation of protein:DNA complex samples, design of a microfluidic flow cell incorporated with OT, automation of OT-confocal scanning measurements, and the development and implementation of a streamlined data analysis package for force and fluorescence spectroscopy data processing. Its components can be adapted to any commercialized or home-built dual-beam OT setup equipped with a confocal scanning microscope, which will facilitate single-molecule force–fluorescence spectroscopy studies on a large variety of biological systems.

## Introduction

Over the past three decades, single-molecule techniques have evolved into versatile approaches for probing the fundamental mechanisms of various biological processes. Single-molecule force spectroscopy techniques, including optical tweezers (OT), magnetic tweezers (MT), and atomic force microscopy (AFM), are widely used to quantify the mechanical properties of biomolecules and monitor their dynamics involving force or contour length changes.^1−5^ Single-molecule fluorescence imaging techniques, including confocal scanning microscopy and total internal reflection fluorescence (TIRF) microscopy, are able to directly visualize molecules of interest and provide information including their stoichiometry and location.^6^ They are uniquely suited for investigating complex systems with multiple molecules involved, as different molecules can be visualized using spectroscopically distinct fluorophores.

**Figure 1:**
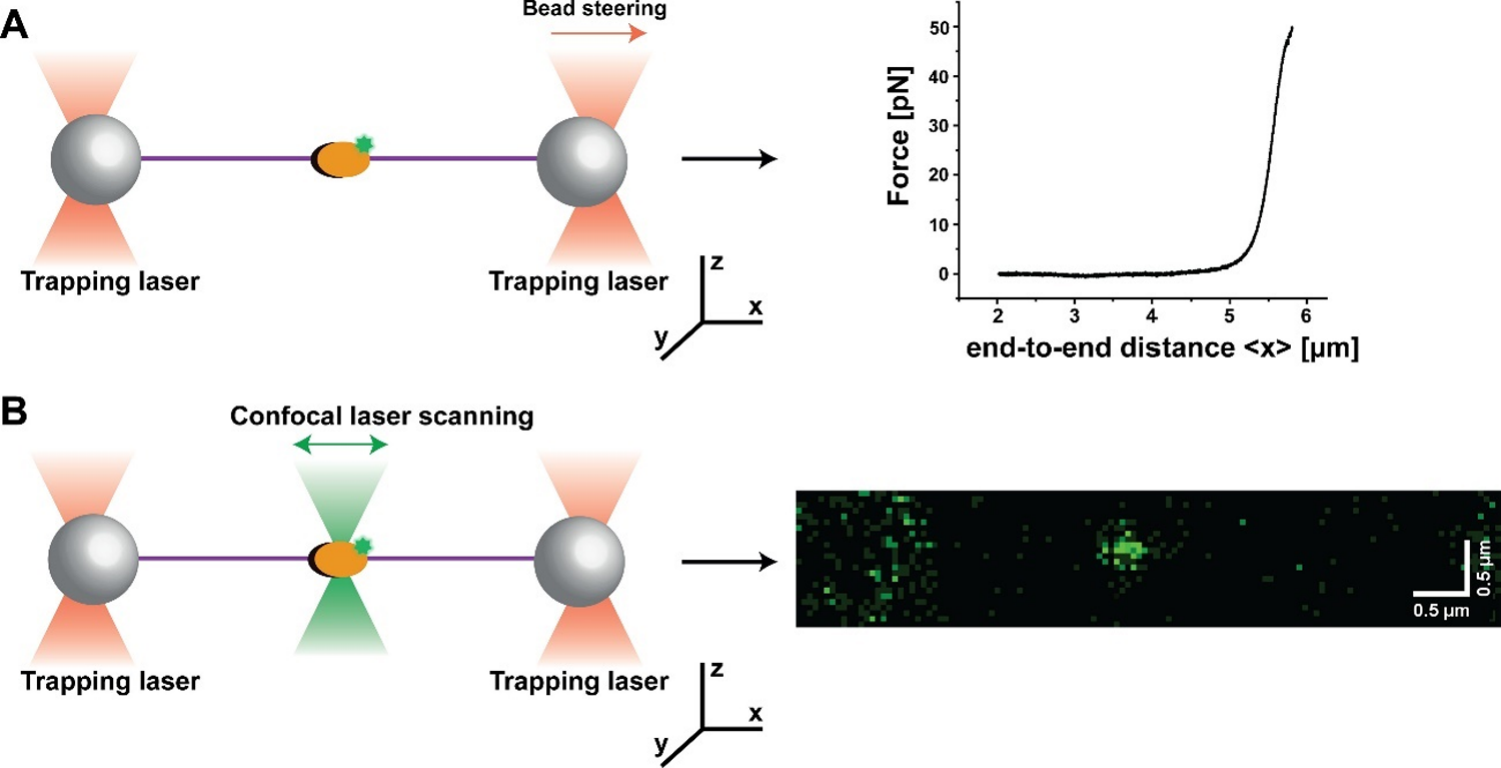
Dual-beam OT setup for force–fluorescence spectroscopy measurements. An individual DNA molecule (purple) is tethered between two micrometer-sized beads (gray spheres) that are held in optical traps (red) to act as a substrate for a fluorescently labeled molecule of interest (orange oval with green dot). A: (left) One of the beads is moved away from the other along the *x*-axis in order to exert a stretching force *F*_*x*_ on the tethered DNA molecule. (right) One then records force–distance data by plotting this force against the end-to-end distance of the DNA measured along the *x*-coordinate. B: (left) A confocal scanning laser (green) integrated with a dual-beam OT performs a line scan along the *x*-axis. (right) A series of offset line scans imaging the *x*–*y* plane to form a 2D image, where a fluorescently labeled CMG holo-helicase^23^ is shown as a fluorescent spot. This allows one to capture the diffraction-limited spots of fluorescently labeled molecules bound to the tethered DNA molecule and monitor their dynamics.

It is highly desirable for researchers to combine force and fluorescence spectroscopy techniques to gain comprehensive insight into complex biological systems. Such integrated force–fluorescence microscopy setups have been realized using AFM,^7,8^ MT,^9−12^ single-beam OT,^13^ and dual-beam OT,^14,15^ in combination with confocal scanning or TIRF microscopy. Among these techniques, dual-beam OT has unique advantages when implemented with fluorescence imaging. In a dual-beam OT setup, two beads are trapped by laser beams that propagate along the (optical) *z*-axis (Figure 1). The beads then tether an individual biomolecule between them in the *x–y* plane. Typically, the fluorescence excitation laser beam also propagates along the *z*-axis (Figure 1B). Therefore, the tethered molecule is perpendicular to both the trapping and the excitation laser beams. In contrast, the orientations of molecules immobilized by MT or single-beam OT typically align with the *z*-axis or have a substantial component along it. For the most commonly used imaging techniques (confocal scanning and TIRF), it is more convenient to image in the entire *x*–*y* plane, rather than to scan along the *z*-axis; furthermore, the increased size of the point-spread function along the *z*-axis relative to the *x*–*y* plane reduces resolution in this dimension.^16^ Therefore, the use of dual-beam OT integrated with fluorescence imaging allows for the convenient extraction of highly resolved information over the entire biomolecule. An additional advantage of dual-beam OT over MT and single-beam OT is that instead of being tethered on the surface at one end, the molecules of interest are separated from the surface by several micrometers, effectively inhibiting undesirable surface adhesion. Taken together, dual-beam OT force-fluorescence spectroscopy has become a powerful single-molecule biophysics technique, broadly used to investigate a number of biological processes including, but not limited to, DNA replication, DNA repair, and protein folding dynamics.^3,17−22^ The focus of this method article is the application of dual-beam OT force–fluorescence spectroscopy in studying protein:DNA interactions, one of the fields that have benefited most from the development of this approach.

As shown in Figure 1A, a dual-beam OT setup typically generates force spectroscopy data by moving one bead in the *x*-direction away from the other bead to apply stretching forces to a DNA molecule immobilized between the beads. The forces are recorded and plotted against the end-to-end distance of the DNA measured along the *x*-coordinate.^3,17^ Such force–distance curves can be subsequently fitted with elasticity models or transformed into contour length space to extract mechanical information about the DNA molecule and/or the molecules bound to DNA (orange oval in Figure 1).

In studies of protein:DNA interactions, dual-beam OT can be integrated with confocal scanning microscopy (Figure 1B) to image fluorescently labeled proteins bound to the trapped DNA and monitor their dynamics.^15,24,25^ A confocal scanning microscope benefits from a very small detection volume (on the order of femtoliters^26,27^) and is able to reject the fluorescence signal outside of the detection volume. This allows for a relatively high concentration of labeled proteins (on the order of ∼10 nM^25,28^) in the measurement solution to monitor their dynamic interactions with DNA.

In this paper, we will discuss several experimental and data analysis aspects required to obtain reliable biophysical data using dual-beam OT integrated with confocal scanning microscopy. First, we will discuss sample preparation strategies suited for investigating complex biological systems, in particular protein:DNA interactions, using dual-beam OT integrated with confocal scanning microscopy and a multichannel microfluidic system that enables well-controlled and flexible sample handling on the microscope. We will then focus on the theoretical background, development, and implementation of a carefully designed analytical tool to extract reliable spatiotemporal, stoichiometric, and mechanical information from dual-beam OT-confocal scanning microscopy data. In doing so, we refer to scripts that we have developed to enable automatic data acquisition and storage on an integrated dual-beam OT-confocal scanning microscope that significantly enhances experimental throughput and reproducibility.

It is worth noting that while the aforementioned analysis tools were developed based on investigations of protein:DNA interactions carried out using the commercially available Lumicks C-trap instrument (https://lumicks.com/products/c-trap-optical-tweezers-fluorescence-label-free-microscopy/), they can be easily adapted to a large variety of experiments carried out with any dual-beam OT-confocal scanning microscopy systems.

## Data Acquisition

### Sample Preparation for Investigating Protein:DNA Interactions

**Figure 2:**
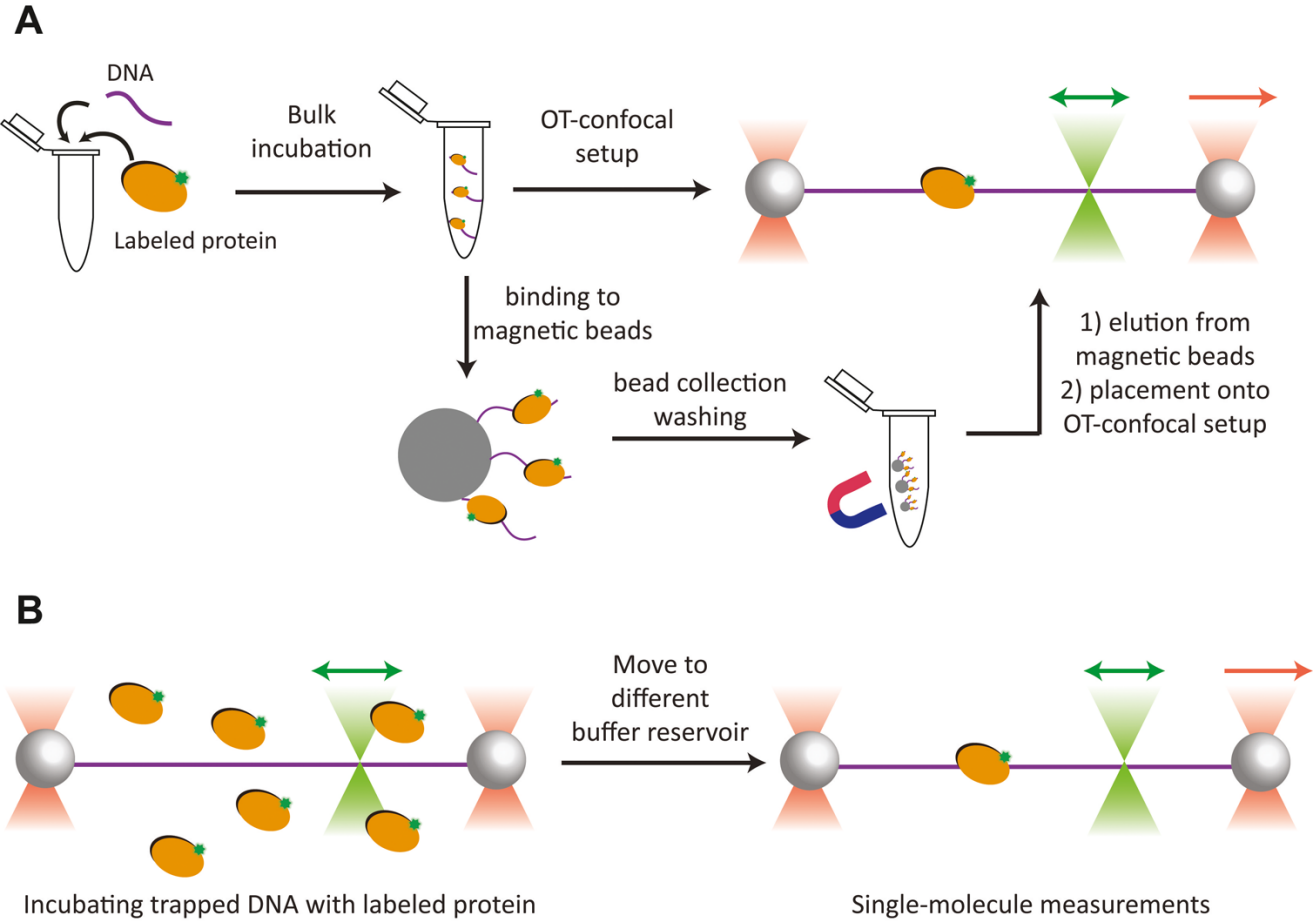
Strategies to prepare protein:DNA substrates. (A) Protein of interest is bound to a DNA substrate through bulk incubation. The assembled protein:DNA complex can be either directly loaded into the dual-beam OT-confocal scanning microscope setup for single-molecule force–fluorescence measurements or purified in bulk prior to single-molecule measurements. (B) Individual DNA substrate molecule is trapped in the dual-beam OT and incubated with a fluorescently labeled protein of interest to form a protein:DNA complex. The complex can subsequently be steered into a separate buffer reservoir for single-molecule measurements in the absence of background fluorescence.

One of the prerequisites for the DNA substrate in dual-beam OT-confocal scanning microscopy measurements is that they should be relatively long. This ensures a sufficient distance between the fluorescently labeled proteins of interest and the edges of the beads, thus, rejecting the noise signal from the beads generated by additional DNA molecules and proteins bound to the beads. The relatively long trap–trap distance also helps to avoid interference between optical traps. Different strategies have been developed to prepare such long DNA substrates, including the use of λ phage DNA^29,30^ or plasmid DNA,^31,32^ synthesis via PCR amplification,^33,34^ and de novo synthesis of plasmid DNA.^35^ Prior to single-molecule experiments, the DNA substrates are linearized if necessary, and their ends are functionalized to allow them to be tethered to the beads. Widely used functional groups for nucleic acid immobilization include small molecule–protein pairs such as biotin:streptavidin, digoxigenin:antidigoxigenin, and to a lesser extent, fluorescein:antifluorescein. These binding groups are highly specific and allow for mechanically stable noncovalent interactions with high affinity.^36−39^ The next step is to bind the proteins of interest to functionalized DNA molecules. This can be achieved either in bulk or at the single-molecule level, or using a combination of both (Figure 2).

The primary advantage of prebinding the proteins of interest in the DNA through bulk incubation prior to single-molecule measurements (Figure 2A) is specific to proteins that require a complex reaction or long incubation with the DNA substrate to function. This is more practically achieved in the bulk context than in the context of a dual-beam OT setup, which is limited in terms of trapping duration and throughput. Another advantage of prebinding is that the resulting protein:DNA complex can be purified, if necessary, once the proteins are bound, thereby removing free protein that could otherwise aggregate onto the DNA and complicate its manipulation (Figure 2A). This is illustrated by our recent work^19^ in which we prebound the DNA substrate onto streptavidin-coated magnetic beads via desthiobiotin:streptavidin interactions and reconstituted the origin-based assembly and activation of the replicative helicase CMG in bulk.^40^ Different washing steps were used to remove unbound protein and aggregates from the magnetic bead-bound protein:DNA complexes, which were subsequently eluted from the magnetic beads using an excess of biotin. We took advantage of the orthogonality between biotin:streptavidin and digoxigenin:antidigoxigenin interactions to then trap the protein:DNA complexes on OT using antidigoxigenin-coated polystyrene beads.

While prebinding the protein in bulk provides flexibility in forming a protein:DNA complex of interest, it is difficult to monitor the intermediate steps in its assembly. Another strategy is to incubate the proteins of interest with individual DNA substrates at the single-molecule level (Figure 2B). This approach provides excellent control of incubation timing by steering the DNA substrate first into and then out of the buffer containing the protein of interest, enabling measurements of protein association/dissociation kinetics and the observation of early intermediates generated in the reactions involving protein:DNA interactions. However, should protein aggregates form, it is more challenging to remove them by washing the protein:DNA complex trapped between the beads because the beads prevent the dissociation of proteins from the DNA ends.

These two approaches can also be combined for complex systems with various proteins involved. For example, in our recent publication,^22^ we prebound histones on double-stranded DNA in bulk to assemble nucleosomes, which requires overnight salt gradient dialysis. Another protein complex of interest, the eukaryotic DNA replication initiator, origin recognition complex (ORC), was subsequently bound onto the DNA by incubating such a trapped single DNA in a buffer containing the ORC.

**Figure 3:**
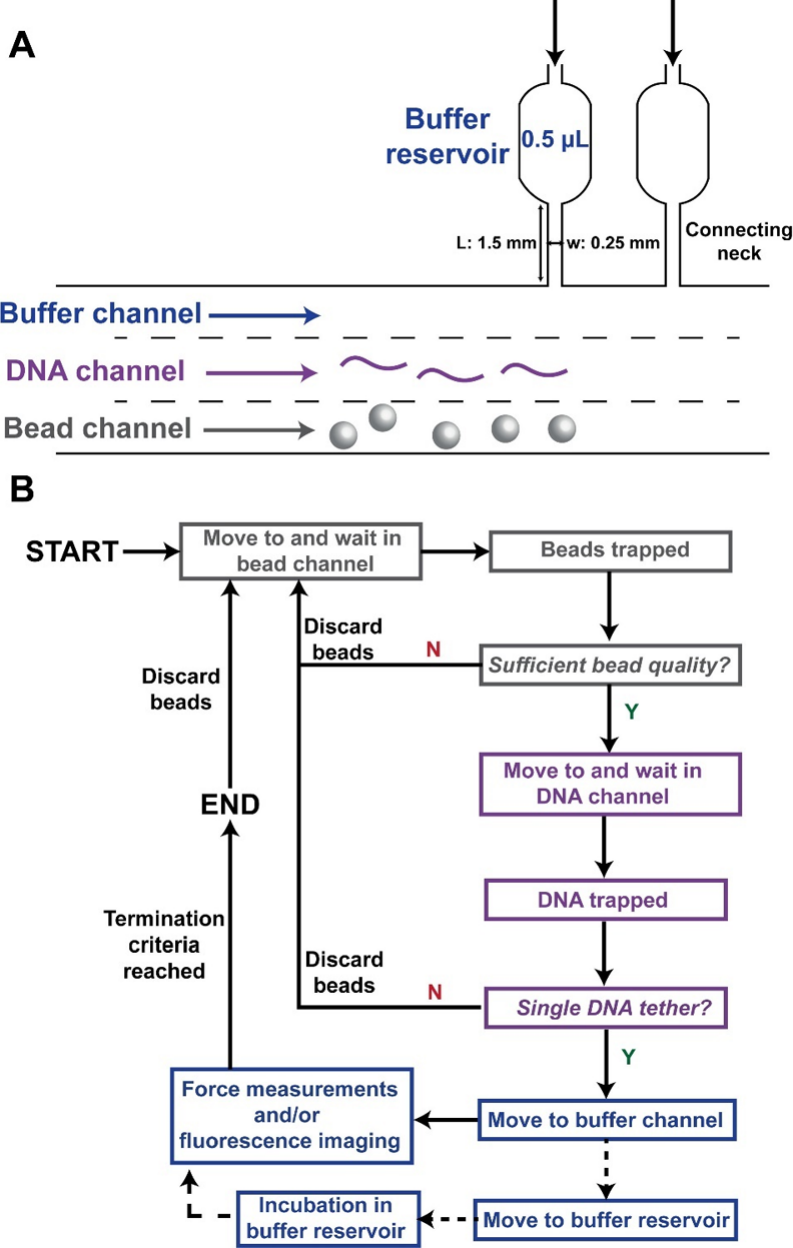
Microfluidic system design and procedure of automated dual-beam OT force–fluorescence microscopy measurements. (A) Microfluidic chip designed to carry out dual-beam OT force–fluorescence microscopy measurements. The bead, DNA, and buffer channels are separated by laminar flow without a physical barrier. Multiple large buffer reservoirs (0.5 μL) are connected to the buffer channel by thin (0.25 mm wide) and long (1.5 mm long) necks. These necks ensure separation between the reservoir and the main channel in the absence of flow. (B) Flowchart showing the procedure for performing automated force and/or fluorescence spectroscopy measurements on an individual DNA molecule. The procedure starts by moving the two trapping laser foci to the bead channel in order to trap beads. Once beads of sufficient quality are trapped, they are steered to the DNA channel to tether a single DNA molecule. The qualities of beads and tethered DNA are monitored as indicated, and not passing the checkpoints leads to a restart of the protocol. Dashed lines show that the movement to the buffer reservoir and subsequent incubation are optional, depending on the experiment. Force spectroscopy and fluorescence imaging are carried out in the buffer channel or a separate buffer reservoir. When measurement termination criteria, e.g., fluorescence intensity or stretching force, are reached, the procedure is restarted to trap and measure another DNA molecule. This procedure is repeated until the desired replicate number of DNAs is reached, and the experiment is terminated.

To implement the aforementioned two approaches, we designed a microfluidic chip system (Figure 3A) that is able to handle very small volume (∼100 μL) of samples with low concentrations (down to ∼10 pM) and provide compartmentalization to separate beads, nucleic acids, proteins, and different buffers. This flow cell is reusable and can be cleaned followed by surface passivation using BSA and pluronics, within ∼4 h. Alternatively, better passivation could be achieved by overnight incubation with passivation reagents. The details of the microfluidic chip design are described in Supplementary Method 1.

In the next sections, we will describe a fully automated data acquisition pipeline suited for the aforementioned sample preparation methods and microfluidic system, which significantly facilitate dual-beam OT-confocal scanning microscopy measurements.

### Motivation and Code Structure for Automated Data Acquisition

A dual-beam OT setup can trap only DNA molecules one at a time. The procedure of trapping beads, trapping DNA, and conducting force spectroscopy and/or fluorescence imaging measurements is repeated for every molecule measured. Conventionally, the measurements are carried out by the experimenter using external input devices such as a mouse, keyboard, or joystick. The repetitive and extensive human input limits both the experimental throughput and the reproducibility of the measurements. Automation of the data acquisition helps address these difficulties by minimizing human input and additionally frees the researchers from repetitive operations on the instruments. In our group, automated data acquisition can increase experimental throughput of certain force–fluorescence spectroscopy measurements by up to 2-fold.

A few scripts are already available on public repositories (https://harbor.lumicks.com/scripts) to automate basic operations of OT, including catching beads, trapping DNA, and carrying out force spectroscopy measurements. We integrated these existing functions with custom-written scripts that enable the automatic collection of confocal scanning data, thereby forming a complete and automated data acquisition pipeline (Figure 3B) that can be directly used on the Lumicks C-Trap system with the aforementioned microfluidic chip (Figure 3A). The code repository consists of three parts that we describe in turn: beads and DNA trapping, confocal scanning, and force spectroscopy. A separate *parameters.yml* file is used to input user-specified parameters.

### Automated Beads and DNA Trapping

The measurements always start by moving the trapping laser to the bead channel and turning on the flow. On the C-Trap system, the trapped beads are assigned a matching score based on the similarity between the bright field image of the bead and a template image preset by the user. Beads with a matching score below the threshold (*bead_match_threshold,* typically set to 90 out of 100) are discarded, as they are likely to be multiple beads trapped in a single trapping laser focus.

Once two beads with matching scores exceeding the threshold are trapped, they are steered to the DNA channel with the flow kept on. DNA molecules with both ends functionalized are brought in contact with the bead surface by the flow, and initially, only one end of the DNA is attached to the beads. As shown in Figure S1A, the flow direction is from the lefthand side to the righthand side. Here, we focus on the DNA molecules with one end attached to the left-hand bead (Bead 1), which are stretched by the flow. The unrestricted end of the DNA is brought close to the righthand bead (Bead 2), which is repeatedly approached to and moved away from Bead 1 along the *x*-axis. Once a DNA molecule is successfully tethered between the beads (Figure S1B), the rightward movement of Bead 2 generates tension on the DNA, resulting in a restoring force on the beads that is detected once it exceeds a preset threshold (*force_threshold*). The flow is then turned off, leaving the remaining DNA molecules with only one end attached to the beads in a collapsed coiled conformation (Figure S1C).

The trapped DNA is subsequently steered to the buffer channel or a buffer reservoir (Figure 3). Incubation in the buffer reservoir may be carried out depending on the experiment, e.g., for binding proteins to trapped DNA (Figure 2B). Prior to fluorescence imaging and/or force spectroscopy measurements, it is necessary to check whether a single DNA molecule is tethered, which is accomplished by comparing the end-to-end extension of the DNA at a specific force with the expected value predicted by the extensible worm-like chain (eWLC) model.^41^ While the eWLC model is limited in its applicable force range (5–30 pN), it is convenient to implement in the code and suitable for most experimental conditions. The final step prior to measurements is to ensure that the DNA is maintained at constant values of the *y*- and *z*-coordinates in the lab frame over its entire length (Figure 1). This alignment ensures that both the forces applied to the DNA and the movement of fluorescently labeled proteins along it have only *x*-components, which significantly simplifies subsequent data analysis.

### Automated Confocal Scanning Imaging

In automated continuous confocal scanning measurements, it is helpful to monitor the fluorescence intensity of the fluorophores and stop the measurement once the fluorophores of interest are bleached as this avoids collecting unusable data. For this purpose, the fluorescence intensities are constantly extracted and projected against the *x*-axis of the image (Figure S2). Once the mean intensities of the relevant fluorescence signals fall below the respective threshold values set by the user (*intensity_threshold*), the confocal scanning measurement is terminated, and the image data is saved. It is worth noting that the images typically contain the beads at the ends of the tethered DNA molecule. This spurious signal from the beads should be excluded from the analysis through the specification of a bead margin (upper panels in Figure S2A, B).

### Automated Force Spectroscopy Measurement

Typical constant pulling speed force spectroscopy measurements are carried out by steering one bead away from the other along the *x*-axis at a constant speed, which results in an increasing stretching force on the tethered DNA molecule. The pulling speed as well as the initial and final end-to-end extensions of the DNA can be specified by the user. In addition, it is also possible to collect repeated forward–reverse force–distance traces on a single DNA molecule until either a preset replicate number is reached or the DNA tether is broken. Such forward–reverse measurements are particularly useful in studying protein folding dynamics.^17^

## Quantification of Confocal Scanning Images

### Data Visualization Methods

**Figure 4:**
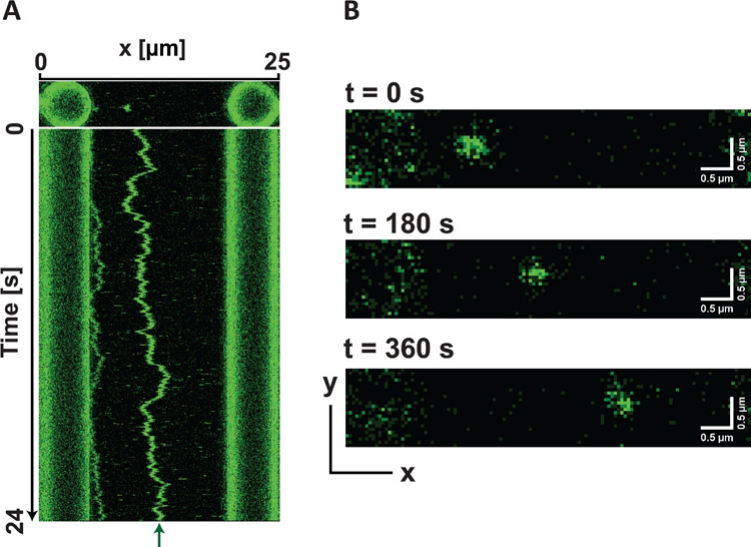
Examples of kymograph and 2D full confocal scanning images collected using dual-beam OT-confocal scanning microscope. (A) A kymograph illustrating the diffusive motion of a fluorescently labeled Mcm2-7 helicase on DNA (green trace indicated by the green arrow). The top panel shows a full 2D scan at the start of the measurement that includes the beads and the fluorescent Mcm2-7 helicase. The kymograph is constructed by repeating 1D scans along a given line on the *x*-axis and stacking the scans (Figure S3). (B) Three 2D confocal scanning images that sample the *x–y* plane at the time stamps are indicated. The green spot illustrates the unidirectional translocation of a fluorescently labeled CMG holo-helicase^23^ on DNA oriented along the *x*-axis.

Kymography and full confocal scanning are two commonly used approaches to visualize the dynamics of biomolecules monitored by confocal scanning microscopy. Kymography creates a single image with which to visualize a dynamic process by making a stack of the line scans acquired in the confocal scanning area at consecutive time intervals (Figures 4A and S3). This provides an overview of the fluorescent intensities along a line collected over time that benefits from the fast imaging of line scans and the attendant high-time resolution. Therefore, kymographs are particularly useful for visualizing rapid motion dynamics of proteins on DNA.^42−44^ However, for in-depth fluorescent spot motion analysis (discussed in the Motion Analysis section), it is important to localize the fluorophore with high precision. The localization precision of fluorescent spots can be measured by calculating the standard deviation of localizations of a static fluorophore (Figure S4). As kymographs consist of only 1D line scans, their localization precision is limited as a result of the loss of information relative to that of the 2D space. In our dual-beam OT force–fluorescence spectroscopy setup, full 2D confocal scans (Figure 4B) yield a ∼2-fold higher localization precision compared to 1D scans (Figure S4), at the expense of reduced time resolution and increased motion blur. Due to these limitations, 2D confocal scans are most suitable to monitor processes at subsecond or slower time scales. A simulation method to quantify the effect of spot velocity on motion blur is discussed in Supplementary Method 2. Because one of the main interests of our confocal scanning data analysis pipeline is the motion analysis of fluorescently labeled proteins bound to DNA, whose accuracy benefits from increased localization precision, the data and analyses presented in the following sections are all based on full 2D confocal scans.

To maximize the insight achievable from tracking the positions of labeled proteins bound to DNA in the instrument, it is necessary to determine the exact correspondence between the fitted pixel position of a fluorophore in a confocal scanning image and its genomic coordinate along the DNA. To achieve this, we must first locate the ends of the DNA in the confocal scanning image and then determine the appropriate conversion of pixels in this image to micrometers and subsequently to kilobases (kbs).

Starting with the first point, a priori it would seem possible to determine the locations of the DNA ends directly from the bead images visible in the 2D confocal scans (Figure 4B); however, the spurious fluorescence signal from the edges of the beads makes it challenging to pinpoint their edges precisely and introduces uncertainty into the location of the ends of the DNA molecule. Fortunately, this difficulty can be overcome by determining the bead positions in the bright-field images, where fluorescence signal is not detected. To then map these positions onto the confocal images, we need to quantify the shift between the two sets of images, which will be discussed in the next section.

### Quantification of Brightfield to Confocal Offset and Confocal Scanning Image Pixel Size

**Figure 5:**
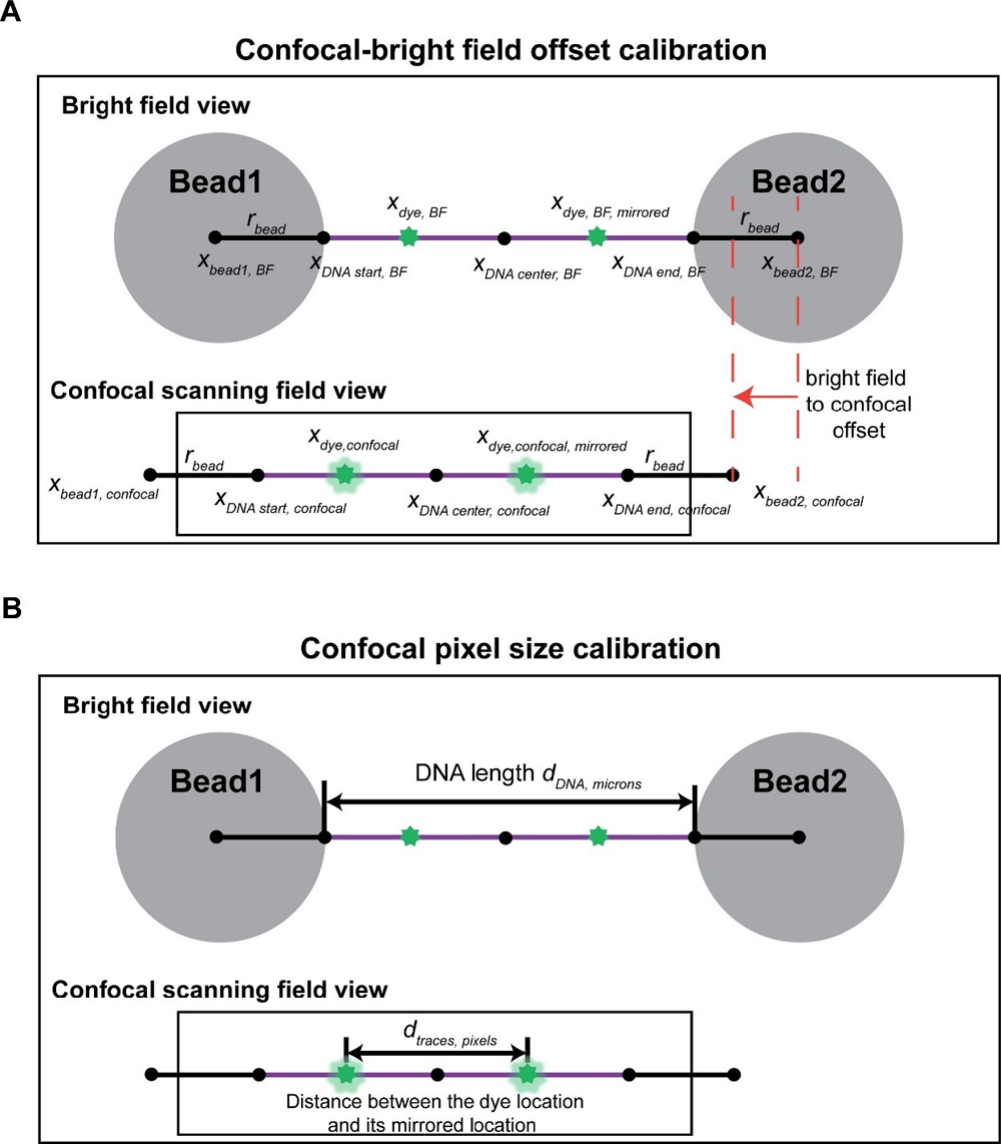
Use of site-specific fluorophores to determine the genomic locations of fluorescent spots. Determination of the bead extremity and pixel-to-nanometer conversion in the confocal scanning images is required for determining the locations of DNA ends.(A)The location of the bead extremity in the bright-field image is mapped onto the corresponding location in the confocal scanning image by calibration of the offset between these two sets of images (indicated by the red dashed lines and arrow). To do so, a static fluorophore is bound to the DNA at a known distance away from the DNA center. This fluorophore will appear at two *x*-locations in the confocal scanning image (green dots, *x*_dye,confocal_ and *x*_dye,confocal, and mirrored_). The arithmetic mean between these two locations yields the center of the DNA in this image (*x*_DNA center, confocal_). Note that the fluorophores are not visible in the bright field image. Therefore, the center of the DNA in the brightfield image is given by arithmetic mean between the bead locations . The offset equals the difference between the DNA center locations in bright field and confocal scanning images. (B) Dividing the distance between the locations of the two fluorophores in the population of traces measured in the confocal scanning images (in pixels) by the length of the DNA measured in the bright-field image (in microns) yields the confocal scanning image pixel size.

To quantify the brightfield to confocal offset, we start by collecting a confocal scanning image data set on a DNA molecule that includes a statically bound fluorescent protein (green dots in Figure 5A) located at a known and unique sequence distinct from the DNA center. This can for example be achieved using a fluorescently labeled dCas9^19,45^ bound to a specific and unique sequence on the DNA. Because the DNA molecule can be randomly trapped in two opposite orientations in a dual-beam OT-confocal scanning microscope, the collected confocal images will display fluorescent spots in two locations, which we designate *x*_dye,confocal_ and *x*_dye,confocal,mirrored_ (Figure 5A). Consequently, the center of the tethered DNA molecule in the confocal image, *x*_DNA center,confocal_, is given by . Conversely, under the assumption that the two optical traps have identical stiffnesses and the beads trapped are equal in size, the center of the DNA molecule in the brightfield images, *x*_DNA center, BF_, is given by (Figure 5A). In consequence, the offset in the *x*-coordinate between the brightfield and confocal images in micrometers is given by
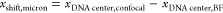
1

We note that the direct readout of the confocal scanning image is in pixels, which nominally have a size (in nanometers) that is set when carrying out the confocal scanning measurements. However, the actual pixel size may differ from this nominal size due to image distortion, and therefore, it must be calibrated. To do so, we first determine the distance in pixels for a given stretching force applied on the DNA, *d*_traces,px_(*F*)_,_ between the oppositely oriented fluorescent spots described in the previous section. As the genomic coordinates of the underlying fluorescently labeled DNA-bound proteins are known, this distance is also known in kbp and designated as *d*_traces,kbp_ (Figure 5B). Dividing these two quantities by each other yields a force-dependent pixel-kbp conversion factor:
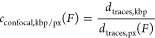
2

Additionally, we know the total length of the DNA in kbp, *d*_DNA,kbp_, and, from the brightfield image, its end-to-end extension in microns, *d*_DNA, micron_, for a given applied force on the DNA (Figure 5B). This yields a force-dependent micrometer-to-kilowatt conversion factor:
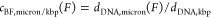
3

Combining these two quantities, we obtain an overall micron-pixel conversion factor, i.e., the corrected pixel size in microns, which is not force-dependent, as the pixel-kbp and micron-kbp conversion factors are determined at the same force applied on the DNA:
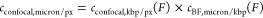
4

With the image offset and pixel size conversion factor *c*_confocal,micron/px_ known, we can calculate the *x*-coordinates of the edges of the DNA in the confocal image. The bead locations *x*_bead1,BF_ and *x*_bead2,BF_ in microns are known from the bright field image (Figure 5A), and the bead radius is taken to be a constant, which is a reasonable assumption given typical vendor specifications, e.g., polystyrene particles from Spherotech Inc. (https://www.spherotech.com/pol_par.htm). The DNA start and end locations in the confocal image can be calculated as follows:
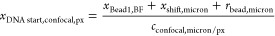
5
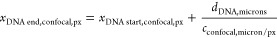
6

So finally, for a pixel location measured in the confocal image *x*_confocal,px_, the corresponding location on the DNA in base pairs can be calculated using
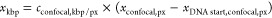
7

The aforementioned calibration parameters are stored in a file named config.yml and an offset correction/tracking parameter file named params_offset_tracking.yml, and are listed in Tables S1 and S2. A full list of optional parameters is provided in the code documentation.

## Confocal Scanning Data Analysis

### Analysis Input and Internal Data Hierarchy

The input data of our confocal scanning data analysis pipeline include (1) multiframe.tiff image data that consists of intensities (ADU, integer values) for three colors at every pixel location and (2) metadata associated with the images. The contents and format of the metadata file are specified in the code documentation.

**Figure 6:**
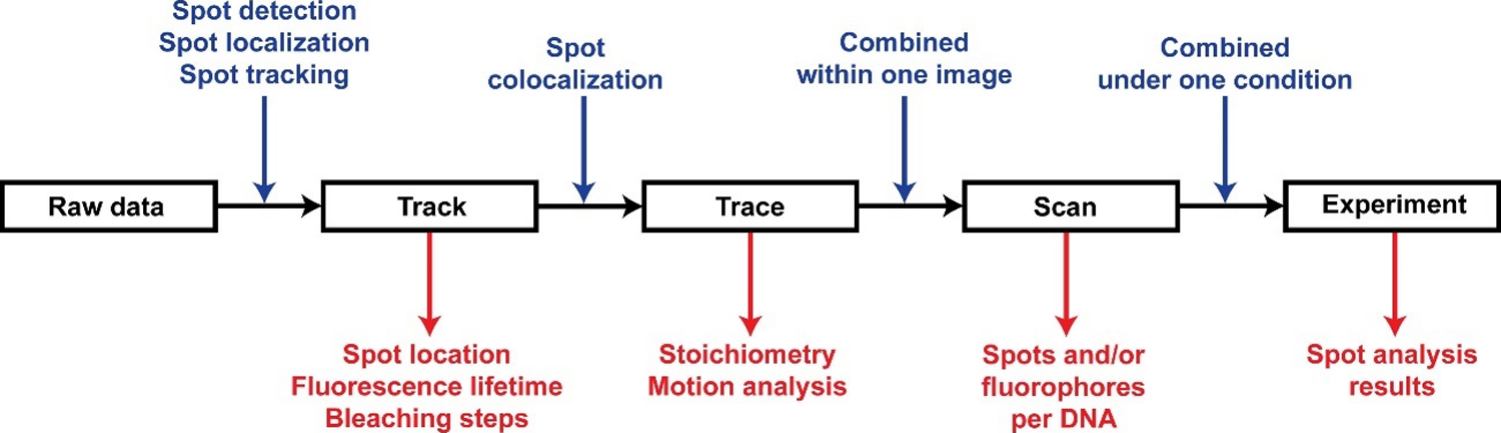
A flowchart summarizing the data hierarchy of confocal scanning data analysis pipeline and the output at each level. The analysis performed to move to the next level are shown in blue. The information extracted at each level is shown in red. The input data include the raw image data and the associated metadata. Spot detection, localization, and tracking algorithms are used to identify fluorescent spots in the image (Track) of a certain color, providing information about locations, lifetime, and number of bleaching steps of the fluorophores. Colocalized spots (Tracks) of different colors are combined within one Trace, which provides information about stoichiometry and motion properties. Different traces within one image are stored in the same scan object, providing information about the number of spots and/or fluorophores on one DNA molecule. All the scans collected under one experimental condition are stored in one experiment object, where the spot analysis results are reported in a table.

The analysis pipeline extracts the following information from the input data: (1) detection of fluorescent spots, (2) tracking of the motion of fluorescent spots in multiframe images, (3) colocalization of spots in different colors, and (4) stoichiometries of different colors in fluorescent spots. The aforementioned information is stored in a hierarchical set of classes to keep track of spots with different colors. This set of classes consists of four levels that are designated Track, Trace, Scan, and Experiment, and summarized in Figure 6.

The most low-level class is the Track class, which stores information about a tracked spot of a single color, such as a numpy array of the *x*-locations in kbp (Track.x_kbp) at each time point in seconds (Track.time_s). Colocalized Tracks are stored in an upper-level class called Trace. In other words, a Trace might contain multiple Tracks of different colors which are at the same location (within the colocalization distance) on the DNA. All of the Traces within one confocal scan image are stored in a Scan object. With the Scan object, we can calculate the number of fluorescent spots as well as the number of fluorophores on the DNA. Finally, the highest-level class, Experiment, contains all Scans that were collected under the same experimental conditions. The technical details of how to detect, track, and analyze spots are explained below.

### Detection, Localization, and Tracking of Fluorescent Spots

For detecting spots in 2D confocal scanning images, we use the Laplacian of Gaussian (LoG) “blob detector” implementation from skimage (https://scikit-image.org/docs/stable/api/skimage.feature.html#skimage.feature.blob_log). A LoG detector has two user inputs, σ and *T*_LoG_. The detector convolves the image with a Gaussian kernel with standard deviation σ, after which a Laplacian operator is applied. This results in a strong response for blobs with a radius , where *r* is the expected point spread function (PSF) radius. This radius can be set for each color separately and should be approximately half of the wavelength. We then look for such responses in the transformed image, selecting any local maxima above the threshold parameter *T*_LoG_.

After detecting the fluorescent spots using LoG, we next determined the subpixel location of each detected spot by fitting its intensity profile to a 2D Gaussian function with a background noise term (Supplementary Method 3). The overall fluorescence intensity of a spot is calculated by summing the intensity values for all of the pixels that lie within the expected PSF radius *r*, minus the background noise.

For the tracking of fitted spots, the user inputs two values: a maximum frame-to-frame linking distance and a maximum frame skip value for connecting track segments. In general, these parameters are picked by considering the expected diffusion coefficients and fluorophore blinking rates. The linear assignment problem (LAP) framework^46^ is used to find out the links between fluorescent spots in different frames. First, track segments are found by listing all possible frame-to-frame connections between fitted spots as elements in a matrix, after which a LAP solver (in our case, scipy’s linear sum assignment optimizer) is used to find the combination of connections with the lowest cost. Then, another such matrix is made for connections between track segments, using the same solver to find connected segments, resulting in full tracks. All spots that are found to be in the same track are given the same *track_id* values in the output table. At this stage, we do not allow for track splitting and/or merging.

The spot detection, localization, and tracking results can be displayed in two ways:

*Full location plots* show all spot detections over time, with connections between spots to indicate tracks.

*Histograms of initial locations* show spot locations fitted with 2D Gaussian on the *x*-axis and the corresponding counts on the *y*-axis. Here, we take the mean location of each trace in the first five frames. Bin sizes are usually chosen taking into account the uncertainty in fluorescent spot localization (this section and Figure S4) as well as the uncertainty in the localization of the DNA ends (Quantification of Confocal Scanning Images section). The trapped DNA has two possible orientations, and in some experiments, it is not possible to know this orientation a priori. In this case, a fluorescent molecule bound to a specific sequence on the DNA has two possible locations on the confocal scanning image that are symmetric with respect to the DNA center (Quantification of Confocal Scanning Images section). For such experiments, it makes more sense to plot the distance from the DNA center along the *x*-axis.

### Fluorescence Lifetime and Bleaching Traces Analysis

Subsequent analysis of tracks includes fluorescence lifetime analysis and determination of stoichiometry.

*Lifetime analysis*: for each frame, we count the number of fluorophores that are still present in all of the tracks in an experiment. This time-vs-fluorophore count data can be fitted with a single exponential decay function, yielding a fitted value for the mean lifetime, or with multiple exponential functions (where the Bayesian Information Criterion (BIC) can be used to determine the number of components), yielding distinct mean lifetimes per component.

*Stoichiometry determination*: a well-established fluorophore stoichiometry measurement method is to illuminate the fluorophores until photobleaching, plot the fluorescent intensity over time, and count the number of bleaching steps.^47−49^ To detect bleaching steps, we use change-point analysis (CPA) as implemented in python library ruptures. In CPA, we minimize a cost function that includes a penalty term for introducing a step (a “change-point”). There are a number of cost functions that one can choose from we use the least squared deviation (CostL2) to detect the shifts in the mean value of the fluorescence intensity in these bleaching traces.

This approach requires two user parameters: a minimum plateau length (in frames) that sets the minimum number of data points that should be in a plateau between two steps and a minimum step size Δ*I*_min_, which can be determined using fluorescently labeled dCas9 (Supplementary Method 4). We set the CPA penalty to Δ*I*_min_^2^ × log (*N*_sig_), where *N*_sig_ is the number of data points in the signal. This formula comes from the BIC (see also eq 30 in ref.^50^).

After CPA with the given minimum plateau length and penalty term, it is still possible to find steps with values below the minimum step size if these steps are deemed significant according to the BIC. This could occur, for example, because a spot moved slightly out of focus, a fluorophore in the background bleached, or a neighboring spot bleached, causing the signal to decrease slightly but permanently. These incorrectly identified steps are filtered out by *pruning*: we eliminate any steps smaller than the minimum step size and set the local fit (between the preceding and following step) to the weighted (by length) average of the preceding and following plateaus.

### Colocalization of Tracks and Crosstalk Correction

Distances between tracks are defined as their average separation over the first five frames of the scan. For track colocalization, we again use the LAP framework, where the cost of colocalizing two spots of different colors is equal to the absolute value of their separation up to a user-specified distance threshold *T*_col_. For distances greater than the threshold, two spots are not considered to be colocalized. If two tracks are colocalized, we call them a trace and they receive the same *trace_id* index in the output table. Quantities like stoichiometry are stored at the trace level. For example, a trace containing a green track with two bleaching steps, and a red track with one bleaching step, has a stoichiometry of (2 green +1 red).

To process colocalized spots (fluorophores *f*_1_ and *f*_2_ with main signals in detection channels *c*_1_ and *c*_2_, respectively), CTrapPy automatically corrects their intensities by subtracting the noise caused by crosstalk between different channels of fluorescence signal. For example, to correct the intensity in channel 2 for crosstalk from channel 1, we use the relationship *I*_*f*2,*c*2,corr_ = *I*_*f*2,*c*2_ – *I*_*f*1,*c*1_ · *c*_*f*1,*c*1→*c*2_, where *c*_*f*1,*c*1→*c*2_ is the crosstalk correction factor (i.e., the relative amount of leakage from channel 1 into channel 2 for fluorophore *f*_1_). This correction can be performed for any combination of channels.

### Motion Analysis

In measurements requiring long imaging time (typically >10 min), drift in the spot location over the measurement might occur. To correct the drift, a control experiment is conducted by imaging a static fluorescent spot on the DNA (Figure 5) and computing the average velocity of this static spot *v*_drift_. This average velocity can be used to correct the spot locations in measurements with moving fluorescent spots: *x*_corr_(*t*) = *x*_raw_(*t*) – *v*_drift_ · *t*. After drift correction, CTrapPy performs different types of motion analysis.

*Processivity* is simply the trace end location minus the trace start location; a processivity plot is a histogram with processivity on the *x*-axis and trace counts on the *y*-axis.

*Diffusion calculations* are done following previously reported approach.^51^ For each trace, the mean squared displacement (MSD) is computed, and the diffusion coefficient is calculated by fitting an optimized number of MSD data points. The baseline for classifying a spot as diffusive can be established by running a static spot data set (e.g., dCas9) through diffusion analysis; the average diffusion coefficient over all static traces gives a baseline value for comparison to other tracks.

*Instantaneous velocity analysis* yields a fitted value of the instantaneous velocity at each time point of every trace of interest. Calculating it directly using *v* = Δ*x*/Δ*t* would result in very wide velocity distributions as a result of the noise present in the location measurements. Instead, we perform a CPA fit to detect linear segments in the velocity (again using ruptures, with the “CostLinear” cost function). This is a way to remove noise and estimate the instantaneous velocity; it also shows how often a trace exhibits velocity changes. Technical details of instantaneous velocity calculation are explained in Supplementary Method 5.

*Anomalous diffusion analysis* can be performed on spots that exhibit significant motion, i.e., spots with a maximum instantaneous velocity above a certain threshold, usually a multiple of a baseline velocity spread in calibration measurements on static proteins. We followed a previously reported method^52^ to perform the anomalous diffusion fit; the anomalous diffusion exponent α is an indicator for motion type (α ≪ 1: subdiffusive or constrained diffusion, α ≈ 1: diffusive, α ≫ 1, superdiffusion or unidirectional motion). Technical details of anomalous diffusion analysis are explained in Supplementary Method 6.

### Spot Analysis Output

The full Experiment object can be exported as a table. Each row contains a detected spot (with an *x*-location, intensity, color, etc.) at a certain frame, with scan_id, trace_id, and track_id. These three indices form a unique identifier for each Track in the Experiment and show which Tracks are colocalized.

Spot tables can be filtered based on stoichiometry, location, and starting frame.

Fluorescent spots (Traces) with large stoichiometries (usually >5 or >10 fluorophore counts, depending on the experiment) are indicative of protein aggregation. These traces are usually not interesting for further analysis and can be filtered out.

If the initial location of a trace is too close to one of the ends of the DNA, this usually indicates that the signals originated from protein bound to the bead and are of no interest for further analysis. Hence, we filter out traces starting too close to the beads.

Finally, in most experiments, we do not expect any proteins to bind to the DNA during the confocal scanning process itself. Hence, traces that start at a later time during this process are indicative of tracking errors. To prevent false spot counts, traces starting at later time frames (usually >5 frames) are filtered out.

The most important columns in the output table are provided in Table S3. The full output specification can be found in the code documentation.

## Force Spectroscopy Data Analysis

As shown in Figure 1A, dual-beam OT force spectroscopy measurements are typically performed by steering one bead away from the other one on the *x*-axis at a constant velocity, thus applying stretching forces to the elastic biopolymer (e.g., DNA) trapped between the beads. Such measurements are mostly used for two purposes: (1) to extract the elasticity parameters, such as contour length and persistence length, of biomolecules^53^ and (2) to detect protein or DNA conformational changes, such as protein folding/unfolding or DNA wrapping/unwrapping in protein–DNA complexes.^54,55^

For the first purpose, we fit the force–distance curves generated by stretching the elastic molecule of interest with elasticity models. Our analysis toolbox provides three elasticity models: the worm-like chain (WLC) model,^56^ the WLC model with enthalpic corrections,^57^ and the eWLC model.^41^ The resulting fitting parameters are persistence length *L*_p,_ contour length *L*_c_, and stretch modulus *S* (for the eWLC model). The parameters of each force–distance curve and a statistical summary are recorded and can be exported.

For the second purpose, we have developed a contour length increment analysis tool as protein and DNA conformational changes are typically associated with contour length changes. The force–distance data measured using force spectroscopy are transformed into a contour length space using the eWLC model. For each data point on the force–distance curve, the contour length is calculated and plotted against force. CPA (Fluorescence Lifetime and Bleaching Traces Analysis section) is used to fit the contour length-force plot and detect contour length changes. The contour length increment, i.e., the difference between the mean contour lengths of the plateaus before and after the change, and the force at which the change happens, are recorded and exported in a summary table. Currently, only the eWLC model is available for contour length increment analysis.

## Conclusions and Outlook

The power of integrated dual-beam OT-confocal scanning force-fluorescence spectroscopy measurements has been demonstrated in the investigation of various biological systems.^58−61^ To make the best use of this powerful tool, careful preparation of biological samples, proper experimental procedures, and rigorous data analysis are all crucial. Here, we have presented a versatile data acquisition and analysis pipeline designed for investigating protein:DNA interactions using an OT-confocal scanning microscope. We discussed different sample preparation strategies for assembling protein:DNA complexes in bulk and/or at the single-molecule level and the biological samples for which these approaches are most suitable. In addition, we designed a microfluidic chip that allows long incubation of the trapped nucleic acid substrate in a buffer reservoir well-separated from the other channels without a physical barrier (Sample Preparation for Investigating Protein:DNA Interactions section and Supplementary Method 1). The aforementioned sample preparation and handling methods form the basis of a high-quality data collection. The data acquisition pipeline is further complemented by an experimental automation protocol, which improves experimental reproducibility and throughput. The source code of the automated data acquisition pipeline is available at https://gitlab.tudelft.nl/nynke-dekker-lab/public/ctrappy-automation.

The data processing pipeline that we developed provides a number of functions to extract spatiotemporal information from confocal scanning images, where we focus on the analysis of fluorescent spots in 2D full confocal scanning images. A number of calibration and filtering steps are taken to remove spurious data, and various analysis tools are used to extract information from the collected data and to inform about the dynamics of measured biomolecules. Different uses and approaches for the analysis of force spectroscopy data are briefly discussed. The source code of the data processing pipeline is available at https://gitlab.tudelft.nl/nynke-dekker-lab/public/ctrappy.

The methods discussed in this article have been demonstrated in a number of studies investigating the dynamics of various protein:DNA interaction systems.^19,22,62^ However, it is worth noting that these tools can be adapted in many other research topics using the same technique, including but not limited to protein folding and dynamics of nucleic acid secondary structures. The broad single-molecule biophysics community could also benefit from the data processing tools presented here, as confocal scanning microscopy and force spectroscopy techniques are also widely used in a number of other single-molecule measurements, e.g., AFM or MT single-molecule force spectroscopy.

## Supporting Information Available

Supplementary methods: microfluidic chip design, simulation of blurred point spread function, fitting of the spot intensity profile, fluorophore bleaching step size calibration, piecewise linear motion fit, and anomalous diffusion analysis; automated trapping of DNA in dual-beam OT (Figure S1); projection of fluorescence intensities on the *x*-axis to monitor fluorophore bleaching (Figure S2); construction of a kymograph (Figure S3); comparison of the localization precision of 1D and 2D scans (Figure S4); description of configuration file parameters (Table S1); and description of tracking parameter file parameters (Table S2) (PDF)
