# Supplementary material for: A Biophysics Toolbox for Reliable Data Acquisition and Processing in Integrated Force–Confocal Fluorescence Microscopy
Source: ACS Photonics. 2024 Mar 18;11(4):1592–603. doi: 10.1021/acsphotonics.3c01739 (PMC11027178; doi:10.1021/acsphotonics.3c01739)
Supplement: Supplementary file 1 — ph3c01739_si_001.pdf [file ph3c01739_si_001.pdf]

Supporting Information for:

A biophysics toolbox for reliable data acquisition and processing in integrated force-confocal fluorescence microscopy

Zhaowei Liu[1,#], Edo van Veen[1,#], Humberto Sánchez[1], Belén Solano[1], Francisco J. Palmero Moya[1], Kaley A. McCluskey[1,†], Daniel Ramírez Montero[1], Theo van Laar[1], and Nynke H. Dekker[1,2,3*]

1 Department of Bionanoscience, Kavli Institute of Nanoscience, Delft University of Technology, 2629 HZ Delft, The Netherlands.

2 Clarendon Laboratory, Department of Physics, University of Oxford, Oxford OX1 3PU, UK

3 Kavli Institute of Nanoscience Discovery, University of Oxford, Dorothy Crowfoot Hodgkin Building, Oxford OX1 3QU, UK

# Equal contribution

† Current address: Department of Physics and Astronomy, Pomona College, Claremont, USA.

*Correspondence: n.h.dekker@tudelft.nl

This Supplementary Information consists of:

- Supplementary Methods (pp. 1-5)
- Figures S1-S4 (pp.6-9)
- Tables S1-S3 (pp. 10-11)
- References (p. 12)

Supplementary Methods

1. Microfluidic chip design

In a typical dual-beam OT-confocal fluorescence imaging measurement, the trapping lasers are used to trap two beads, after which one traps an individual DNA molecule between them. The resulting bead-DNA complex is then steered towards a separate compartment to load proteins onto the DNA, and/or to carry out force spectroscopy or fluorescence imaging measurements. To avoid intermixing between the bead, DNA, and buffer solutions, it is highly preferable to maintain them in distinct compartments. A widely used compartmentalization strategy is to create distinct channels using laminar flow (1),(2), as this obviates the introduction of physical barriers (see the bead, nucleic acid, and buffer channels in **Figure 3A**). While highly convenient, this approach does come with the limitation that the flow has to be maintained to ensure separation between the channels during long measurements or incubation. Furthermore, constant flow increases the shear stress on the DNA substrate, which facilitates dissociation of protein:DNA complexes, and can complicate force spectroscopy measurements.

To overcome these issues, we have designed a flow cell with relatively large buffer reservoirs (~0.5 µL) separated from the main channel by a long thin connecting neck (1.5 mm long, 0.25 mm wide, **Figure 3A**) to reduce the rate of diffusion between them. This allows the user to turn off the flow in all channels and carry out more precise imaging and force spectroscopy measurements there. The inclusion of several such reservoirs provides the flexibility to include different storage reservoirs for reagents used in different incubation and/or imaging steps. For example, a protein can be loaded onto the tethered DNA molecule in one reservoir at the single-molecule level (**Sample preparation for investigating protein:DNA interactions** section), and subsequent measurements can be carried out in a different reservoir, if necessary. In our instrument, steering the DNA molecule from one reservoir to an adjacent reservoir by moving the sample stage relative to the trapped beads takes ~30 s (moving at a speed of 0.2 mm/s). At this movement speed, the drag force applied by the surrounding fluid on the beads (1.7 µm diameter) is estimated to be around 3 pN, which is sufficiently low to present minimal influence on the stability of the beads in the OT and the DNA molecules trapped between them.

2. Simulation of blurred point spread function

To quantify the motion blurring occurring in 2D confocal scan images, we have simulated the scan process based on the following parameters:

Scan length L [px]	Scan width H [px]	Pixel size [nm]	Pixel time t [ms]
124	24	50	0.2

Our simulations show that the blurring produces the elongation (in the moving direction of the fluorescent spot) and rotation of the PSF function. These two quantities are our metrics to determine the motion blurring. We use a rotated Gaussian fitting function to measure them:

$$g\,(x,y) = Aexp[-a(x - x_0)^2 + 2b(x - x_0)(y - y_0) + c(y - y_0)^2] + B$$

$$a = \frac{1}{2}(\frac{cos^2\theta}{2\sigma_x^2} + \frac{sin^2\theta}{2\sigma_y^2})$$

$$b = \frac{1}{2}(\frac{sin2\theta}{4\sigma_y^2} + \frac{cos2\theta}{4\sigma_x^2})$$

$$c = \frac{1}{2}(\frac{sin^2\theta}{2\sigma_x^2} + \frac{cos^2\theta}{2\sigma_y^2})$$

Here, A represents the amplitude, B the noise background, (x_o, y_o) the center coordinates, (σ_x ,σ_y) the standard deviations, and θ the rotation angle.

For example, we simulated the unidirectional movement of a fluorescent spot at 1.7 nm/s (corresponding to a eukaryotic replicative DNA helicase) (3), which gives rise to negligible motion blur. In contrast, a fast moving fluorescent spot (200 fold faster, 340 nm/s), gives rise to a clearly rotated Gaussian point spread function, as shown in the table and figures below.

v [nm/s]	σ [px]	σ_y [px]	σ_x [px]	θ [deg]
1. 7	3	3.0015	2.9986	-
340	3	3.3195	2.7149	41.93

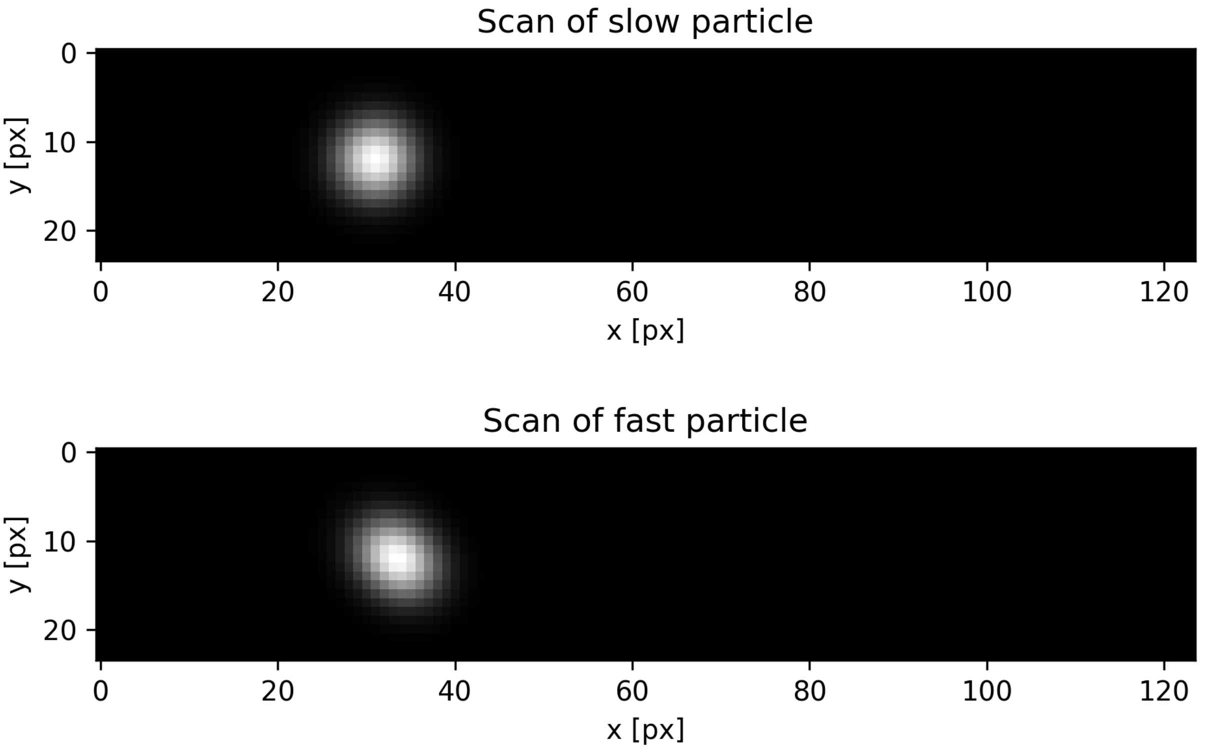

3. Fitting of spot intensity profile

The intensity profiles of detected fluorescence spots are fitted by 2D Gaussian function with a background term:

$$g\,(x,y) = \,Aexp\left[-\frac{(x\,-x_0)^2}{2\sigma_x^2}-\frac{(y\,-y_0)^2}{2\sigma_y^2}\right]+B$$

where the fitted parameters are:

A: The amplitude (intensity value corresponding to the mean possition) of the detected spot.

(x_o, y_o): The x and y coordinates of the detected spot, respectively.

(σ_x ,σ_y): The standard deviation in x and y of the detected spot, respectively.

B: The intensity correction due to background noise.

The estimated intensity value of any spot corresponds then to the sum of photons in a circular mask (given a detection radius), taking into account the background noise B.

4. Fluorophore bleaching step size calibration

To calibrate the minimum and average bleaching step size (ΔI_{min} and ΔI_{avg}, respectively) of a fluorophore, we typically singly label dCas9 with this fluorophore, attach the labeled dCas9 to a unique location on the DNA molecule through the use of an appropriately selected guide RNA, and continuously image the dCas9-bound fluorophore until it bleaches. In this case, provided that the fluorophore is well-behaved, the overwhelming majority of fluorescent spots should exhibit one bleaching step. Quantification of its associated mean intensity provides an excellent basis for starting the calibration process. The user inputs an initial guess for the minimal step size ΔI_{min} for the dCas9 dataset, after which CTrapPy generates fitted intensity plots and a step size distribution graph. The step size distribution has a mean ΔI_{avg} and a standard deviation $\sigma_{\Delta I}$. The user updates the initial guess until two conditions are met:

- A large majority of the fitted intensity plots look good upon visual inspection;
- $\Delta I_{min} \leq \Delta I_{avg} - 2\sigma_{\Delta I}$, meaning that at least 95% of bleaching steps will be larger than the minimum step size.

In the case of multi-color measurements, it is important to check the crosstalk between different channels, and correct the fluorescence intensity taking the crosstalk into consideration. The method to correct for crosstalk is described in **Colocalization of tracks and intensity correction** section.

5. Piecewise linear motion fit

First, a Kalman filter is applied to the spot locations of each trace using the pykalman Python library, in order to reduce noise. The resulting locations are fit to linear segments using CPA, with a linear cost function, to fit multiple linear segments. The minimum segment size and penalty term can be set by the user After the fit, every detected spot has an associated fitted velocity v_{CPA}, which has lower noise than an instantaneous velocity calculated by simply subtracting subsequent spot locations. Of course, the piecewise linear fit only makes sense for static traces (with or without drift), and traces exhibiting piecewise linear motion.

The mean velocity μ_v of v_{CPA} for all traces in the static calibration dataset can be used for drift correction, using $x_{corrected}(t) = x(t) - t \cdot \mu_v$. This drift correction is not necessary if $t_{image} \cdot \mu_v \ll \sigma_x$, where σ_x is the location error. The standard deviation σ_v of the velocity distribution can be used as a cutoff to determine if a spot is static or is undergoing motion.

6. Anomalous diffusion analysis

After MSD analysis on the drift-corrected traces, we can fit an anomalous diffusion exponent α. This exponent gives us a characterization of motion type for each trace. We start with:

$$MSD(\tau) = D_\alpha \tau^\alpha + 2\sigma_x^2$$

where D_α is the anomalous diffusion constant, τ is the lag time and σ_x is the location error. $\alpha \ll 1$ corresponds to spots exhibiting confined diffusion; for diffusing particles we expect $\alpha \approx 1$; $\alpha \gg 1$ indicates particles undergoing unidirectional motion. The actual fit of α is done using the formula

$$log(MSD(\tau) - 2\sigma_x^2) = log(D_\alpha) + \alpha log(\tau^\alpha),$$

so that we can use linear regression. We use least squares to fit up to a maximum lag time corresponding to one third of the length of the actual fit. After that, the MSD generally exhibits fluctuations that are too large to perform reliable fitting.

Supplementary Figures

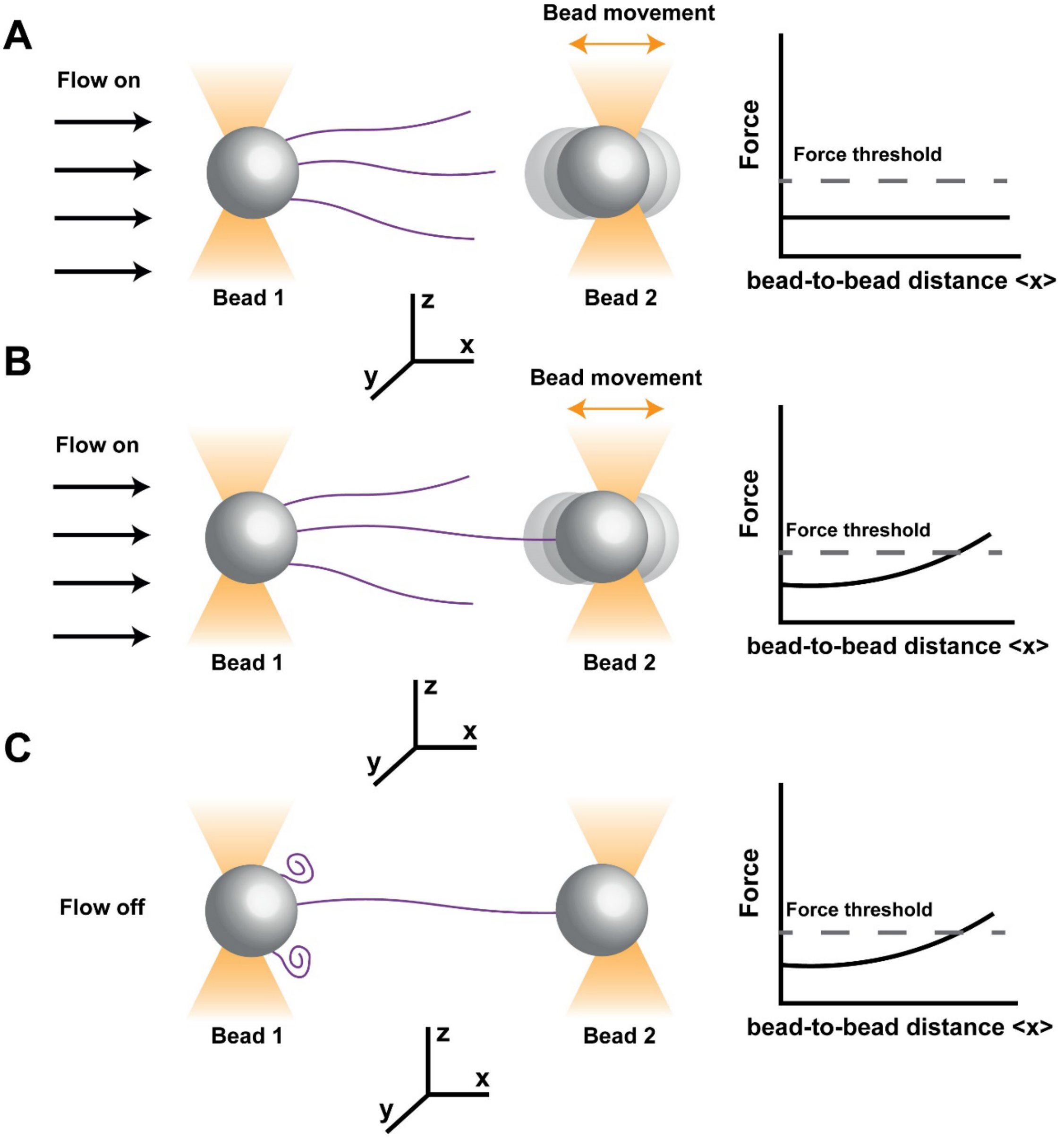

Figure S1 Automated trapping of DNA in dual-beam OT. A: Functionalized DNA molecules are brought in contact with the beads by the flow to have one end of the DNA attached to the beads. Those DNA molecules attached to Bead 1 are stretched by the flow and the other end of the DNA is brought close to Bead 2 by the flow. Bead 2 is constantly moved close to and away from Bead 1 on the *x*-axis. The force exerted on the beads is low and independent of the bead-to-bead distance when the DNA is not tethered between the beads. **B:** Once the other end of the DNA is attached to Bead 2, rightward movement of Bead 2 generates a force that exceeds a force threshold set by the user, indicating that one or more DNA molecules are tethered between the beads. **C:** The flow is then turned off, leaving the remaining DNA molecules attached to the beads in a coiled and collapsed conformation.

A

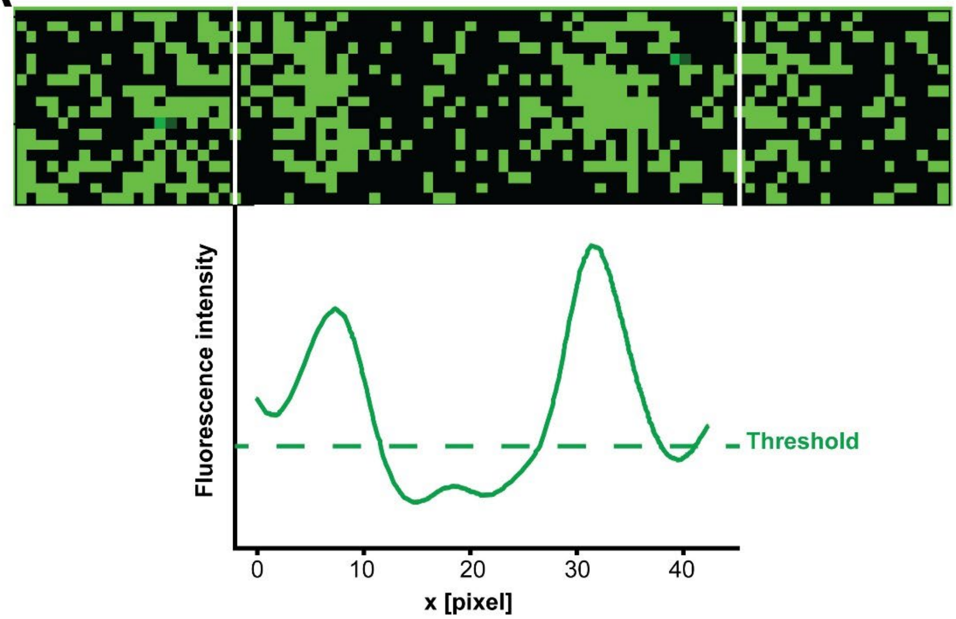

B

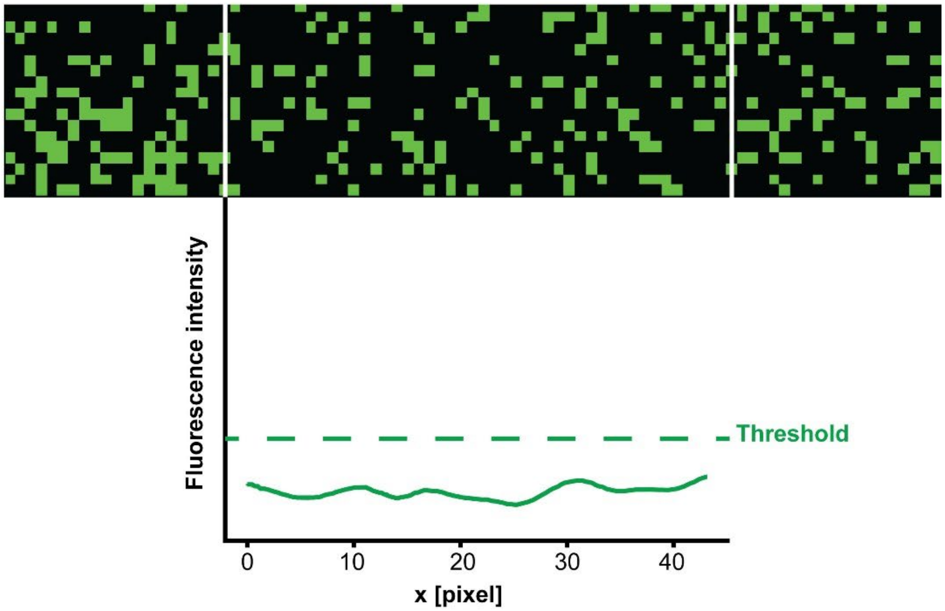

Figure S2 Projection of fluorescence intensities on the x-axis to monitor fluorophore bleaching. A: Two fluorescent spots are present in the 2D confocal scanning image (upper panel), giving rise to two peaks on the fluorescence intensity projection plot (lower panel) exceeding the preset intensity threshold. The signals close to the beads (beyond the white lines on the upper panel) are excluded from the analysis by specifying a bead margin. **B:** After the fluorophores bleach, the background fluorescence intensity falls below the threshold. The continuous confocal scan is subsequently terminated.

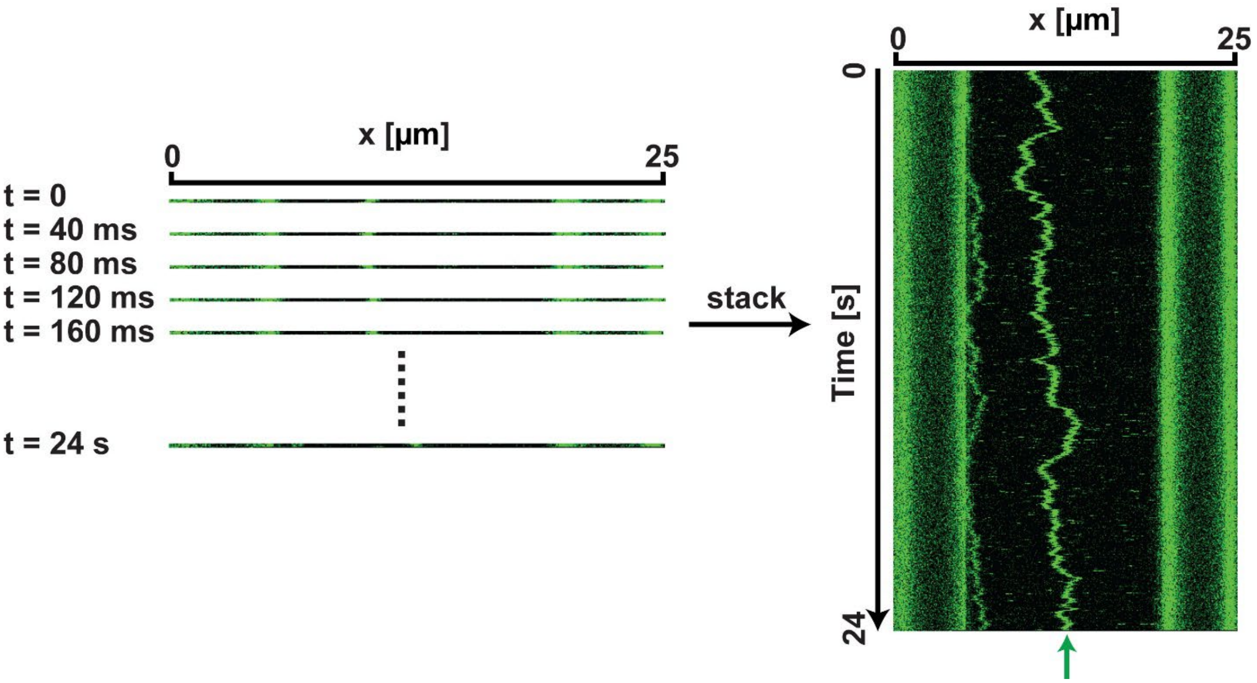

Figure S3 Construction of a kymograph. (left panel) 1D scans are repeated along a selected line along the *x*-axis. A kymograph is constructed by stacking these 1D scans to form a visual representation (right panel) in which one axis is the *x*-axis and the other axis represents time. The motion of the fluorescent molecule over time is shown by a green line (indicated by the green arrow in the right panel).

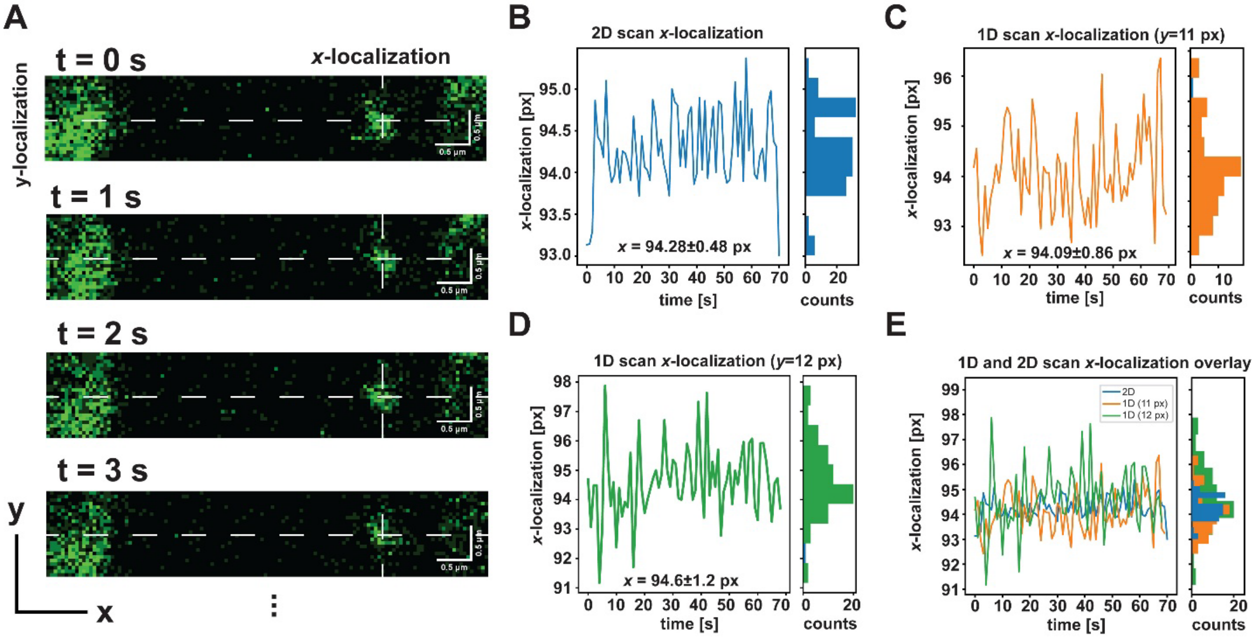

Figure S4 Comparison of the localization precision of 1D and 2D scans. A: A fluorescently labeled DNA oligo is hybridized to a single-stranded flap on a double-stranded DNA trapped between two beads to form a static fluorescent spot in the confocal scanning image. The fluorescent spot is detected by continuous 2D confocal scans with a pixel size of 50 nm and a frame rate of 1 s/frame until the fluorophore bleaches. The *x*- and *y*- localizations of the spot are indicated by the white dashed lines, respectively. **B:** The *x*-localization of the fluorescent spot in each 2D image is determined by 2D Gaussian fitting and plotted against time. Mean and standard deviation of the localization are indicated in the figures. The distribution of the *x*-localization is shown in the histograms at right. **C and D:** The *x*-localization of the fluorescent spot in each frame is determined by 1D Gaussian fitting to a selected line in the 2D confocal scan image (white dashed line in panel **A**) to mimic the line scans used to build kymographs. Two different *y*-coordinates close to the center of the fluorescent spot are selected to carry out this 1D scan analysis. The average locations and standard deviations are shown in the figures. The distribution of the *x*-localizations is shown in histogram. **E:** The time traces and distributions of *x*-localizations of the fluorescent spot determined by 2D images versus 1D scans are overlayed. The standard deviations of *x*-localizations determined by 1D scan analysis are approximately 2 fold higher compared to 2D scan analysis. Therefore, 2D scan analysis yields a ~2 fold higher fluorophore localization precision than 1D scans.

Supplementary Tables

Table S1: Description of configuration file parameters.

Field	Type	Description
`colocalization_distance`	float	Colocalization distance (micron)
`step_counting_divide`	boolean	Do we divide steps by the expected step size?
`confocal_pixel_size`	float	Width of one pixel in confocal image (micron)
`min_step_sizes_{lasers}`	3 integers	Minimum step sizes for r, g, b, for each possible combination of active confocal lasers
`avg_step_sizes_{lasers}`	3 integers	Average step sizes for r, g, b, for each possible combination of active confocal lasers
`bleed_through`	3x3x3 floats	Bleed through matrix. For axes (i, j, k) and value x: laser i causes the j fluorophore to leak x% of their signal into channel k.

Table S2: Description of tracking parameter file parameters.

Field	Type	Description
`offset`	float	Brightfield-confocal shift (micron)
`laser colors`	boolean	Active confocal lasers; 'r', 'g', 'b', 'rg', 'rb', 'gb', or 'rgb'
`thresholds`	3 floats	LoG detection threshold; numbers for RGB separately
`radius (pixels)`	3 floats	LoG detection radius; numbers for RGB separately
`max frame skip`	3 floats	Max time skip for tracking (frames)
`max dist between spots (pixels)`	3 floats	Max distance skip for tracking (pixels).

Table S3: Description of scan table columns.

Column name	Type	Level	Description
scan_id	integer	scan	identifier for scan
trace_id	integer	trace	identifier for colocalized tracks of different colors
track_id	integer	track	identifier for a spot of a single color, tracked over time
color_id	integer	track	identifier for spot color: (0, 1, 2) for ('r', 'g', 'b'), respectively
frame	integer	spot	frame number
x_pixel	float	spot	x-location of spot in pixels
time_s	float	spot	time in seconds
x_kbp	float	spot	x-location of spot in kbp from left bead edge
intensity	float	spot	sum of pixel values in detected spot
corrected_intensity	float	spot	intensity corrected for crosstalk
dna_start_pixel	float	scan	starting pixel coordinate of DNA
dna_end_pixel	float	scan	ending pixel coordinate of DNA
dna_length_kbp	float	experiment	DNA length in kbp
file_name	string	scan	path to tiff file associated with scan
lifetime	integer	track	lifetime of the track in frames
step_count	integer	track	number of steps in track, from step fitting the bleaching trace

References

(1) van Mameren, J.; Modesti, M.; Kanaar, R.; Wyman, C.; Wuite, G. J. L.; Peterman, E. J. G. Dissecting Elastic Heterogeneity along DNA Molecules Coated Partly with Rad51 Using Concurrent Fluorescence Microscopy and Optical Tweezers. *Biophys. J.* **2006**, *91* (8), L78-80.

(2) Candelli, A.; Wuite, G. J. L.; Peterman, E. J. G. Combining Optical Trapping, Fluorescence Microscopy and Micro-Fluidics for Single Molecule Studies of DNA-Protein Interactions. *Phys. Chem. Chem. Phys.* **2011**, *13* (16), 7263–7272.

(3) Kose, H. B.; Xie, S.; Cameron, G.; Strycharska, M. S.; Yardimci, H. Duplex DNA Engagement and RPA Oppositely Regulate the DNA-Unwinding Rate of CMG Helicase. *Nat. Commun.* **2020**, *11* (1), 3713.
